# Supplementary material for: Sunbeam: an extensible pipeline for analyzing metagenomic sequencing experiments
Source: Microbiome. 2019 Mar 22;7:46. doi: 10.1186/s40168-019-0658-x (PMC6429786; doi:10.1186/s40168-019-0658-x)
Supplement: Supplementary file 3 — McCann et al. 2018 report. Report with figures reproducing results from McCann et al. 2018 [64]. (HTML 812 kb) [file 40168_2019_658_MOESM3_ESM.html]

McCann et al 2018: Post-FMT viral taxa


# McCann *et al* 2018: Post-FMT viral taxa

#### *13 January, 2019*

## Background

This report uses the Sunbeam pipeline to reproduce findings from �Viromes of one year old infants reveal the impact of birth mode on microbiome diversity� (PMID: 29761040) by Zuo *et al*. A key finding of this study is that the virome at 1 y/o seems to correlate with the birth mode�that is, that children born by spontaneous vaginal delivery (SVD) have higher viral diversity and higher *Anelloviridae* richness than children born by C-section. The purpose of this report is to see whether analysis with Sunbeam reproduces these results. This report was generated by the extension sbx\_mccann2018; this link also includes instructions for re-running this analysis from the beginning. This report was run using the Kraken Standard database built 1 October, 2018.

## Results

Below are boxplots generated using the `ggplot2` package in R. Each point corresponds to a single sample. We want to see whether we also find greater Anellovirus richness in spontaneous vaginal delivery (SVD) compared to C-section, and greater overall virus diversity in SVD compared to C-section:

### Anellovirus richness

### Virus diversity

## Conclusion

The results of our analysis are comparable to those reported by McCann *et al*. The Wilcoxon rank-sum test p-value (p=0.01102403) is similar to that reported in the paper (p=0.014). While the difference in viral diversity does not reach significance (p=0.1035892)), we do see the same relationship in Shannon diversity (higher diversity in SVD) as McCann *et al*.
